# Oxidative Stress in Reproduction: A Mitochondrial Perspective

**DOI:** 10.3390/biology9090269

**Published:** 2020-09-04

**Authors:** Alexandra Almansa-Ordonez, Raquel Bellido, Rita Vassena, Montserrat Barragan, Filippo Zambelli

**Affiliations:** 1 Eugin, 08006 Barcelona, Spain; aalmansa@eugin.es (A.A.-O.); mbarragan@eugin.es (M.B.); fzambelli@eugin.es (F.Z.); 2 Centro de Infertilidad y Reproducción Humana (CIRH), 08006 Barcelona, Spain; rbellido@cirh.es

**Keywords:** reactive oxygen species, oxidative stress, mitochondria, gametogenesis, assisted reproduction

## Abstract

Mitochondria are fundamental organelles in eukaryotic cells that provide ATP through oxidative phosphorylation. During this process, reactive oxygen species (ROS) are produced, and an imbalance in their concentrations can induce oxidative stress (OS), causing cellular damage. However, mitochondria and ROS play also an important role in cellular homeostasis through a variety of other signaling pathways not related to metabolic rates, highlighting the physiological relevance of mitochondria–ROS interactions. In reproduction, mitochondria follow a peculiar pattern of activation, especially in gametes, where they are relatively inactive during the initial phases of development, and become more active towards the final maturation stages. The reasons for the lower metabolic rates are attributed to the evolutionary advantage of keeping ROS levels low, thus avoiding cellular damage and apoptosis. In this review, we provide an overview on the interplay between mitochondrial metabolism and ROS during gametogenesis and embryogenesis, and how OS can influence these physiological processes. We also present the possible effects of assisted reproduction procedures on the levels of OS, and the latest techniques developed to select gametes and embryos based on their redox state. Finally, we evaluate the treatments developed to manage OS in assisted reproduction to improve the chances of pregnancy.

## 1. Introduction

Mitochondria are multifunctional cellular organelles involved in several aspects of cell biology. Their main function is the production of adenosine triphosphate (ATP) through oxidative phosphorylation (OXPHOS), but they also regulate other cellular processes like calcium homeostasis, fatty acid metabolism and a variety of signaling pathways. Mitochondria are present as multiple units within each cell, in a variable number and structure depending on the size of the cells and their energetic requirements. Two different types of mitochondria can be identified based on their structure and metabolism [1]. Orthodox mitochondria, present in glycolytic cells, have an ovoid form, large matrix volume, small intra-cristae volume and lamellar cristae. In contrast, mitochondria during high respiratory activity generating ATP through OXPHOS are defined condensed, and are characterized by relatively small matrix volume and an expanded intra-cristae space with cristae shaped as crescents. The production of ATP through OXPHOS proceeds via a series of reversible reactions of oxidation and reduction of nicotinamide adenine dinucleotide (NAD+/NADH) or flavine-adenine dinucleotide (FAD/FADH_2_) molecules by different protein complexes (Complex I-IV), that allow the generation of a proton gradient in the intermembrane space of the organelles, which is used by an ATP synthase (Complex V) to convert ADP and organic phosphate into ATP. This process is fundamental to support the high energetic requirements from the earliest phases of development, as it guarantees a much higher yield of ATP compared to glycolysis (production of 36 vs. 2 ATP molecules per glucose molecule metabolized). However, during the subsequent reactions of oxidation and reduction that characterize OXPHOS, reactive oxygen species (ROS) are produced, mainly in Complex I and Complex III, which are responsible for the conversion of Coenzyme Q10 (CoQ10) into ubiquinol [2]. ROS are reactive molecules and free radicals (molecules having one unpaired electron) containing oxygen. The most representative ROS in biology are the hydroxyl ion (OH^-^), the superoxide ion (O_2_^−^) and hydrogen peroxide (H_2_O_2_) [3]. The presence of ROS has been classically considered harmful for the cell, and a disbalanced presence of ROS give rise to a condition called oxidative stress (OS). The concept of oxidative stress (OS) was firstly formulated in 1985 by Helmut Sies and defined as “an imbalance between oxidants and antioxidants in favor of the oxidants, leading to a disruption of redox signaling and control and/or molecular damage” [4].

However, ROS are not always associated to oxidative stress, as their presence is a key factor for several physiological processes, and a depletion of ROS in developmental and somatic cells causes impairments in cellular differentiation, immune response and autophagy [3,5,6].

Now it is clear that finely regulated levels of ROS are required for the correct functioning of the organism, while alterations in the redox equilibrium are detrimental for cells and can cause molecular damages at the level of lipid, protein and DNA.

**Figure 1:**
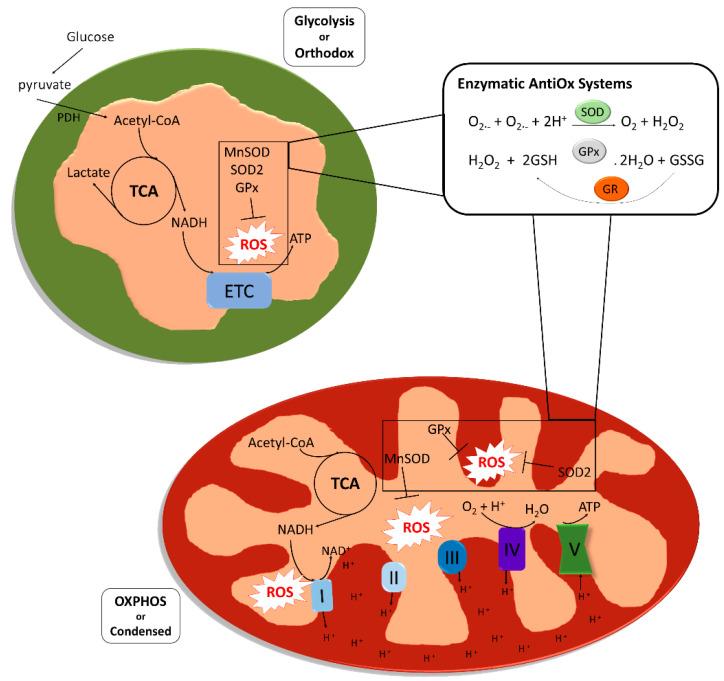
Structure, metabolic activity and reactive oxygen species (ROS) scavenging system in mitochondria. Mitochondria present in glycolytic cells with low oxidative phosphorylation (OXPHOS) rates are defined as “orthodox mitochondria” (colored as green), and are characterized by an ovoid form, large matrix volume, small intracristal volume and lamellar cristae. Mitochondria colored as red, the so called “condensed mitochondria”, are formed to provide cells with adenosine triphosphate (ATP) generated by OXPHOS. Condensed mitochondria are characterized by relatively small matrix volume and an expanded intracristae space with cristae shaped as a crescent. Both orthodox and condensed mitochondria share two enzymatic systems to counteract ROS that are produced in both glycolytic and OXPHOS metabolism. The first antioxidant reaction consists of the reduction of two superoxide anions to hydrogen peroxide catalyzed by a superoxide dismutase (SOD2 or MnSOD). The glutathione peroxidase catalyzes the reduction of the hydrogen peroxide to water oxidizing a glutathione molecule. Glutathione reductase catalyzes the reduction of the oxidized glutathione molecule, thus renewing reduced glutathione reservoir (ATP, adenosine triphosphate; ETC, electronic transport chain; GPx, glutathione peroxidase; GR, glutathione reductase; NAD, nicotinamide adenine dinucleotide; OXPHOS, oxidative phosphorylation; PDH, pyruvate dehydrogenase; ROS, reactive oxygen species; SOD, superoxide dismutase; TCA, tricarboxylic acid).

Although ROS are naturally produced during cellular life, OS can be caused by exogenous sources like environmental factors, smoking or alcohol intake, and from within the cell by a variety of cellular processes like protein synthesis. One of the biggest cytoplasmic sources of ROS is the nicotinamide adenine dinucleotide phosphate (NADPH) oxidases (NOX), required to catalyze electron transfer from NADPH to molecular oxygen to form superoxide [7]. Despite these sources do generate a significant amount of ROS, the majority of ROS seem to be generated via mitochondrial metabolism (Figure 1).

Eukaryotic cells, and specifically mitochondria, are equipped to keep ROS levels under control; it is very important in fact that the organelles have a system for ROS scavenging that ensures that the reactive species do not reach high concentrations and cause damage, or induce apoptosis. Among the enzymes involved in mitochondrial ROS scavenging, superoxide dismutase is one of the most studied. There are five families of primary intracellular antioxidant enzymes: (i) copper/zinc-superoxide dismutase (Cu/Zn-SOD, SOD1) functioning in the cytosol, (ii) manganese-superoxide dismutase (Mn-SOD, SOD2) acting in the mitochondrial matrix, (iii) catalase, (iv) glutathione peroxidase (GPX) and (v) glutathione reductase (GR). SODs catalyze the dismutation of the superoxide ion to oxygen (reduction) and hydrogen peroxide (oxidation), while catalase and GPX convert hydrogen peroxide into O_2_ and disulfide glutathione (GSSG), respectively, and H_2_O. Small molecular weight and nonenzymatic antioxidants are also involved in the protection of the intracellular components against the reactive oxygen species [8]. These species can be classified in two groups based on their solubility: water soluble and lipid soluble. In the first group we can find molecules like the glutathione (GSH) and vitamin C, while molecules like vitamin E and β-carotene are found in the second group. Lipid soluble antioxidant are especially effective preventing lipid peroxidation, while water-soluble antioxidants are mainly involved in potential reduction, they also participate in the regeneration of lipid-soluble antioxidants [9].

In reproductive tissues and gametes, mitochondria are receiving an increasing amount of attention, as their activity is being linked to early gametogenesis and necessary for a proper embryo development. In this context, energy production does not seem the main function of mitochondria, which have a very peculiar activation pattern and a limited production of ATP. The reason for these low metabolic rates is being debated, but different studies show that it may be the need for low ROS exposure [10].

In assisted reproductive technologies (ART), patients receive a non-physiological hormonal stimulation, their gametes are cultured in vitro and the embryos are left to develop for 3–5 days post fertilization inside an incubator. All these processes are known to cause cellular stress to a certain extent, and several researchers tried to evaluate the potential damage produced, and looked for possible strategies to improve ART by intervening on OS management [11].

In this review, we discuss how the mitochondrial metabolism and mitochondrial derived ROS influence gametogenesis, and how this could impact the embryo development in vitro. Finally, we present the current evidence on the effectiveness of testing and selecting gametes/embryos based on OS levels, and the antioxidant/mitochondrial treatments that are being employed to reduce oxidative damage.

## 2. Oxidative Stress and Mitochondrial Activity in Gamete Development

**Figure 2:**
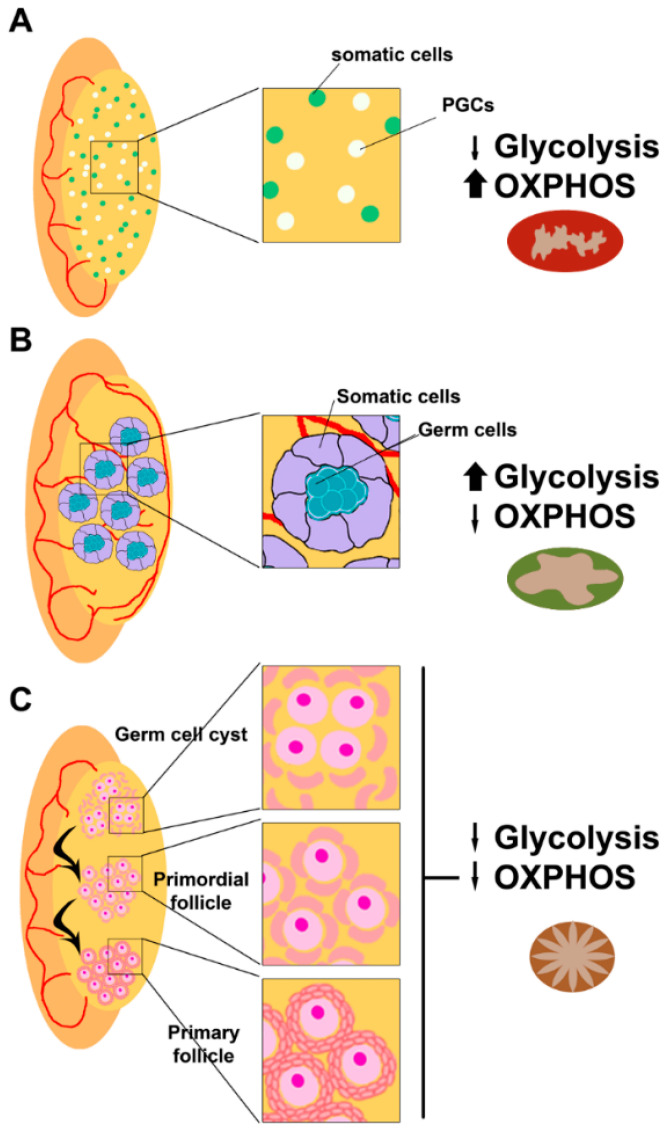
Mitochondrial requirements during fetal gonad development. (**A**) After migration to the genital ridge, primordial germ cells (PGCs in white) colonize the gonadal ridges, and start proliferating and specifying with the help of gonadal somatic cells (in pale green). At that point, mitochondria present at PGCs present a condensed form that corresponds to their metabolic requirement of ATP provided by OXPHOS (colored as red). Sexual commitment depends on the gonadal environment and PGCs commit to spermatogenesis (**B**) in a fetal testis or oogenesis (**C**) in a fetal ovary. (**B**) In a fetal testis, germ cells proliferate to form spermatogonia, which are glycolytic cells presenting orthodox mitochondria (colored as green). At this moment, signals provided from somatic cells of the developing testis block the meiotic entrance of spermatogonia. (**C**) In a fetal ovary, germ cells commit to oogenesis in the absence of inhibiting signals. PGCs proliferate and differentiate to oogonia and undergo meiosis to form primary oocytes. At that moment, the metabolic requirements are low and mitochondria present in those primary oocytes are transcriptionally and bioenergetically silent (quiescent state colored as brown). ATP is almost entirely provided by somatic cells surrounding oogonia and primary oocytes.

Early during embryo development, primordial germ cells (PGCs) are produced just after implantation at the extraembryonic ectoderm, where they receive signals for proliferation and migration to the gonadal ridge [12]. Once the PGCs colonize the gonadal ridges, they start proliferating and specifying [13]. In the male fetus, spermatogonia entry into meiosis is inhibited by signals from the developing testis, while in the female fetus, oogonia undergo meiosis to primary oocytes (Figure 2).

It has been shown in mice that PGCs have different metabolic requirements compared to their embryonic stem cells (ESC) counterparts, increasing their dependence on OXPHOS and glutathione metabolism during differentiation. These data suggest that differentiating PGCs are actively using the machinery for the stress response (glutathione metabolism) against reactive oxygen species (ROS) produced as byproducts of OXPHOS, to maintain cell integrity [14]. However, their role in embryonic gonad development needs further research.

### 2.1. Mitochondrial Metabolism during Spermatogenesis

**Figure 3:**
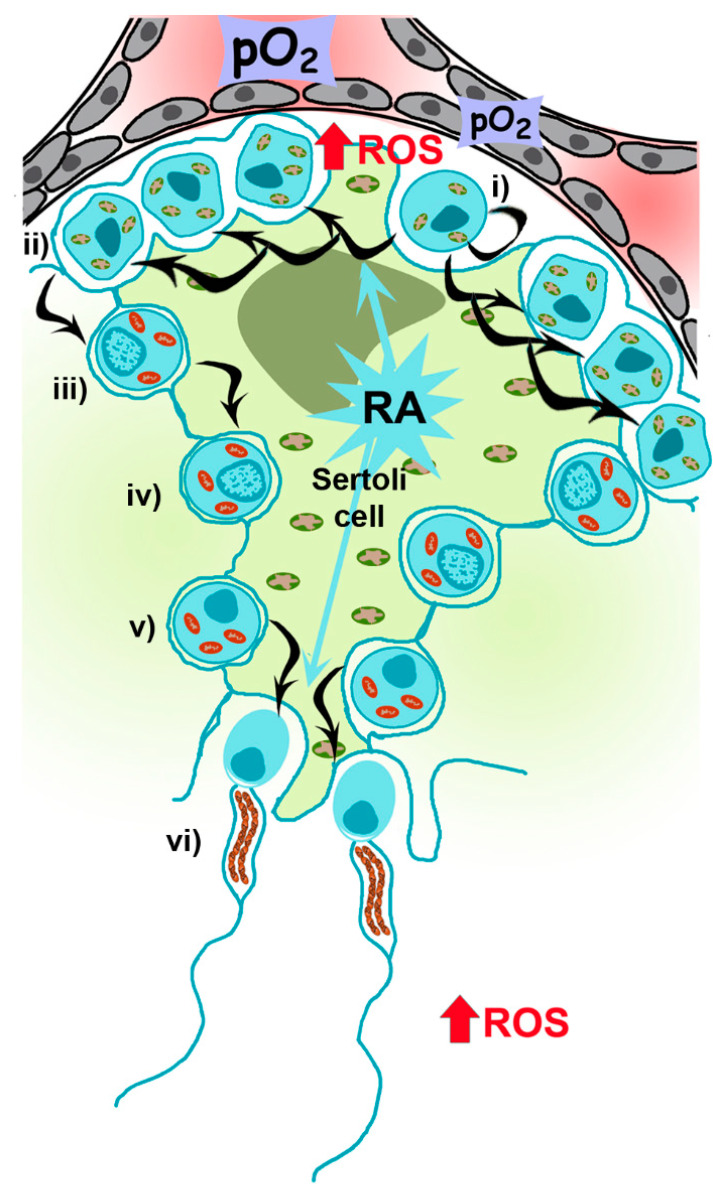
Mitochondrial requirements during spermatogenesis. The spermatogenic process is mainly driven by Sertoli cells (big green cell engulfing the spermatogenic cascade), glycolytic cells carrying orthodox mitochondria, providing and controlling nutritional support to germ cells throughout their development. (**i**) Spermatogonia, proliferating and self-renewing cells, are glycolytic cells presenting orthodox mitochondria and allocated at the basal part of the seminiferous tubule, close to Leydig cells where vascularization is low. Every 60 days, differentiation is engaged by retinoic acid (RA) produced by Sertoli cells, to spermatocyte precursors (**ii**) and up to primary spermatocytes (**iii**), when OXPHOS metabolism becomes the main mechanism to produce ATP (condensed mitochondria, in red). After two meiotic divisions at the spermatocyte stage (**iii,iv**) producing early spermatids (**v**), which need again RA signaling from Sertoli cells to differentiates to spermatozoa (**vi**), with few mitochondria rearranged in the midpiece. Once spermatozoa are produced, they will receive signals from physiological levels of ROS in the epididymis to ensure proper sperm maturation, and in the oviduct to promote hyperactivation and acrosome reaction to allow sperm–oocyte fusion during fertilization.

Mammalian spermatogenesis is an asynchronous, highly dynamic and metabolically active process supported by Sertoli and Leydig (somatic) cells. On average, 30–60 days are required for the differentiation of self-renewing spermatogonia into spermatocytes and, after two meiotic divisions, the formation of four haploid spermatids that differentiate into specialized spermatozoa [15] (Figure 3).

Altogether, a regulated redox state seems to be important to ensure a proper spermatogenic process. During spermatogenesis, the role of mitochondria is not only to provide energy to the sperm, as they are also involved in many other cellular processes such as sex steroid hormone biogenesis, control of the germ cells pool by proliferation/apoptosis balance, ROS production and calcium signaling [13,16].

Spermatogenesis is highly dependent on the metabolism of retinoic acid (RA), a metabolite of vitamin A1 (all-trans-retinol) [17], which acts in a paracrine manner, with Sertoli and germ cells controlling the storage of retinol by esterification [18]. Mice knock-out for the gene encoding for RARα, Rara−/−, exhibit defects in spermatogenesis and morphological abnormalities [19]. Moreover, the reduction of RARα expression in human sperm from patients suffering of varicocele has been recently described [20]. Nevertheless, the mechanisms driving its action on metabolic requirements during spermatogenesis (i.e., regulating mitochondrial number and functionality in spermatocytes and sperm) are not defined yet, although it has been recently reported that RA could induce oxidative phosphorylation and mitochondria biogenesis in adipocytes [21]. Spermatogonia contains mitochondria with orthodox morphology and glycolytic-based metabolism, reflecting the self-renewing and proliferating nature of the cells [22]. Once differentiation is engaged, RA-dependent signaling is active up to preleptotene spermatocytes (primary spermatocytes), when OXPHOS metabolism, a more efficient mechanism to produce ATP, increases [23]. Metabolic changes are accompanied by morphological changes, from ovoid with lamellar cristae (present in spermatogonia) to a more elongated and condensed with cristae shaped as crescents [24,25]. During this transition, there is an increase in mitochondrial DNA (mtDNA) replication, an assembly of respiratory complexes [26] and, as recently reported in mice, an increase in mitochondrial fusion driven by mitofusins (Mfn1 and Mfn2), which acts as a quality control mechanism increasing mitochondrial metabolism [27]. Mfn1 and Mfn2 knockout mice, for instance, are unable to differentiate spermatogonia into spermatocytes. Those results are in concordance with the results shown by Yao and colleagues indicating that cell proliferation requires an increase in OXPHOS supported by mitochondrial fusion [28].

The second part of spermatogenesis involves two meiotic divisions, first from primary spermatocytes to secondary spermatocytes and a second meiotic cycle to produce early spermatids. At this point, Sertoli cells actively re-engage differentiation to spermatozoa (spermiogenesis), once again depending on RA metabolism [17]. This process implies the loss of most of the cytoplasm in residual bodies containing mitochondria. However, during spermatogenesis, the number of mitochondria decreases to 20–80 in the mature spermatozoon, supporting the theory of uniparental (maternal) mitochondrial inheritance [12] and a role of OXPHOS metabolism at the final steps of spermatogenesis. These few mitochondria rearrange at the periphery of tail microtubules in tubular structures anchored around the anterior portion of the nine outer dense fibers (ODFs) and the axoneme, forming the midpiece [29,30] in a complex of filaments called submitochondrial reticulum [31]. This small number of mitochondria is important for male fertility as disruption of these structures reduces fertility in mice [32]. Ultrastructural studies using reducing agents (e.g., dithiothreitol) have shown that the outer mitochondrial membrane (OMM) of sperm mitochondria is covered by a keratinous structure formed by disulfide bonds between cysteine- and proline-rich seleno-proteins [33], such as phospholipid hydroperoxide glutathione peroxidase (PHGPx), which uses hydroperoxides to maintain this structure.

This location suggests a strong biological reason, such as the provision of energy for sperm motility. In addition, and compared to mitochondria from other somatic cells, sperm mitochondria are very specialized and possess specific isoforms of proteins and isoenzymes, which functionally differentiate from the somatic ones [34]. Their specific location and the fact that they have been shown to consume less oxygen than somatic mitochondria, points at the unsolved question of sperm metabolic requirements to generate approximately the same maximum electric potential [14,15] for sperm motility.

### 2.2. Role of Mitochondria and ROS during Spermatogenesis

Different metabolites originate from mitochondria depending on their metabolism. As indicated before, during spermatogenesis mitochondria change their metabolic state from glycolytic to OXPHOS [35] (Figure 3). The relevance of sperm mitochondria may thus be associated with their role in other physiological features, particularly with the production of ROS, which in controlled levels are needed for proper sperm function [36,37].

Physiological levels of ROS participate in crucial events acting as an intracellular signal transducer by taking part in sperm chromatin condensation and in plasma–membrane rearrangements, both happening in the epididymis during sperm maturation [38]. Moreover, ROS are responsible for the phosphorylation of several proteins that participate in hyperactivation, capacitation and the acrosome reaction, promoting these two events [39]. This is reflected by a general bulk of tyrosine-phosphorylated proteins. As an example for capacitation, adenylate cyclase (AC) is activated by Ca^2+^ and·O_2_^-^, producing cyclic adenosine monophosphate (cAMP) and activating protein kinase A (PKA), stimulating NADPH oxidase and generating greater ROS production (specifically H_2_O_2_) and activating protein tyrosine kinase (PTK) to phosphorylate Tyr residues of the fibrous sheath surrounding the axoneme, the cytoskeletal component of the flagellum.

ROS also provide fluidity to the cellular membrane, an essential attribute for sperm–oocyte fusion, by inhibiting the phosphatases that prevent the degradation of fatty acids [40].

Therefore, a balance between ROS and antioxidant defenses is crucial to maintain an optimal sperm maturation and function. Pathological levels of ROS in sperm are accompanied by alterations in vital biomolecules (lipids, nucleic acids, proteins and sugars) [41].

ROS imbalance produces a cascade of reactions in the plasma membrane resulting in lipid peroxidation, causing a loss of 60% of the membrane fatty acids. Consequently, the fluidity of sperm plasma membrane diminishes, enhancing non-specific permeability to ions and inhibiting the function of membrane receptors and enzymes [42,43]. Apart from the plasma membrane, ROS can also cause damage in the mitochondrial membrane allowing the exit of cytochrome C, which activates the apoptotic caspases. This is reinforced by the findings that spermatozoa from infertile men with increased ROS had more levels of apoptosis than spermatozoa from fertile men [42].

An additional layer of ROS regulation of sperm function is represented by the biogenesis of tRNA- and rRNA-derived small RNAs (tsRNA and rsRNA, respectively). These small RNAs have been found to induce epigenetic changes, and cause intergenerational transmission of metabolic disorders [44,45]. This was recently shown in mice, where a high fat diet (HFD) of the father have been associated to the onset of metabolic disorders in the offspring, with a concomitant change in the expression and RNA modifications of a subset of tsRNA in the father sperm [44]. In addition, the injection of tsRNA from HFD mouse sperm into normal zygotes alone can originate metabolic disorders in the offspring, strongly suggesting an active role for this class of RNAs. In humans, the upregulation of tsRNA was also observed in sperm samples obtained after a week of high-sugar diet, hinting to a conserved mechanism [46]. There seems to be a very complex ROS-regulated system in sperm, affecting both sperm functionality and offspring health through epigenetic modifications [47,48].

### 2.3. Mitochondria and ROS in Folliculogenesis and Oogenesis

In several mammalian species, including humans, the development of the ovary includes multiple cell types (oogonia, theca and granulosa cells) and depends on two interrelated processes, folliculogenesis and oogenesis, which include ovarian growth during fetal development and follicular maturation starting at puberty.

Oogonia undergo several rounds of mitotic division with incomplete cytokinesis forming multiple germ cell cysts or germ cell nests [49], while somatic cells present at the nascent ovary proliferate to interact with them [50,51]. Some of the oogonia in the nest undergo cell death, breaking the intracellular bridges, and allowing the somatic cells in the ovary to surround the individual oogonia. Then, the oogonia stop dividing and begin to undergo meiosis to form haploid oocytes and establish the final ovarian reserve. Around birth, meiotic progression in oocytes arrests at the diplotene stage of prophase I and remains at this stage until the oocyte is recruited for ovulation [52] (Figure 2C).

**Figure 4:**
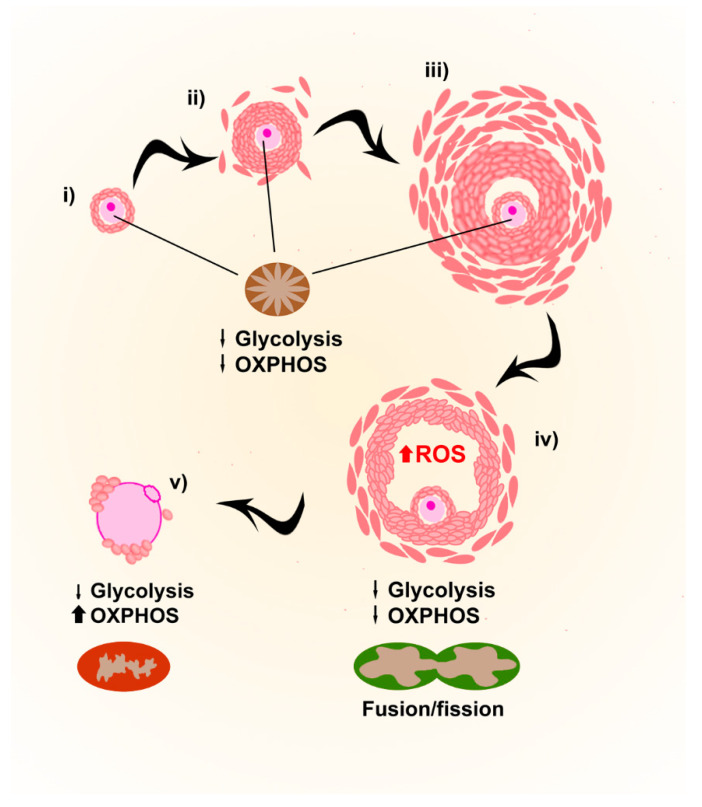
Mitochondrial requirements during oogenesis. After birth and up to puberty, primary follicles (**i**) and immature oocytes are stored in a quiescent state characterized by immature mitochondria. After puberty, folliculogenesis starts producing secondary follicles (**ii**), antral follicles (**iii**) and preovulatory follicles (**iv**). The low energetic requirements of growing oocytes are provided by cumulus cells (theca and granulosa cells), providing ATP and pyruvate by metabolizing glucose via glycolysis. In preovulatory follicles, granulosa cells increase ROS levels to favor apoptosis of cumulus cells to induce ovulation. During final meiotic maturation, just before ovulation (**v**), functional mitochondria are located in a layer in the subcortical region.

During folliculogenesis, the developing oocyte is surrounded by cumulus cells (theca and granulosa cells), which are contributing to several functions for its support and growth, like providing ATP and pyruvate by metabolizing glucose via glycolysis [53] (Figure 4).

Although in most species cytoplasmic organelles (e.g., mitochondria, endoplasmic reticulum and Golgi elements) in oocytes from primordial follicles are uniformly allocated close to the nucleus, in humans they group asymmetrically around the nucleus in Balbiani bodies [54]. The immature and spherical few hundreds of mitochondria present in Balbiani bodies, with a reduced ability to produce ATP, increase their number throughout early stages of oogenesis [55], but points towards low energy requirements for the quiescent oocytes during early stages of development, also due to the energy supply provided by the follicular environment.

During final meiotic maturation, mitochondria in oocytes change their localization, probably as a consequence of the cytoplasmic rearrangements of the cytoskeleton occurring during this phase. In mature MII oocytes, they distribute throughout the cytoplasm, with a layer of polarized mitochondria in the subcortical region (reviewed in [56]). ATP levels increase during polar body extrusion, and higher amounts of ATP have been correlated to higher fertilization rates of mature MII oocytes [47,48]. Mitochondrial fusion and fission, in addition, are fundamental processes during the whole maturation process. Mitofusins 1 and 2 are the proteins responsible for it, and the deletion of either gene in oocytes results in compromised developmental competence with elevated ROS levels [57,58].

### 2.4. ROS (in)Balance in Follicles and Oocytes

Currently, it is accepted that cumulus cells contribute to buffer redox conditions into the follicle (including the oocyte) via metalloproteins. The cytosolic Cu/Zn-SOD and the mitochondrial Mn-SOD, two of the metalloproteins present in eukaryotes, have been found in preantral and antral follicles, up to the stage of the dominant follicle [59,60,61].

ROS levels increase in the final stages of follicle development, and during ovulation, they increase in the granulosa cells of the thin wall of the follicle, inducing apoptosis and leading to a breakage of the wall and the liberation of the cumulus–oocyte complex (COCs) [55]. Additionally, ROS present in the follicular fluid can influence folliculogenesis and steroidogenesis, and is involved in the initiation of apoptosis in antral follicles (recently reviewed by [56]). ROS seems therefore to be important for the regulation of follicular growth, but whether they have an active role or appear as a consequence of the higher metabolic activity during final follicle maturation is not proven yet.

In maturing oocytes, the oxidation of pyruvate for ATP generation is accompanied by the increase of O_2_ intake [62] and therefore ROS production [63,64]. The glutathione (GSH-GSSG) reductase system is stored in the oocyte during maturation, and it is not only used to protect the oocyte against increasing concentrations of ROS, but also to protect the embryo during the initial cleavages [65,66,67]. High concentrations of ROS can induce several defects in the oocyte, including the possible destabilization of the M-phase promoting factor (MPF) and the consequent decrease in survival-promoting factors, ultimately leading to mitochondrial-triggered apoptosis [61].

However, physiological levels of ROS seem to participate in meiosis resumption in the growing oocyte. In small rodents, for example, a decrease in catalase activity and increase of hydrogen peroxide as well as total ROS levels trigger meiotic resumption from diplotene arrest in rat follicular oocytes [68]. Although increasing data on the activity of ROS and antioxidants during oocyte development and maturation were produced in recent years, the identification of conditions for the improvement of ART seem still far away.

## 3. Mitochondria and ROS in Embryos

Mitochondrial metabolism is the main source of energy in mammalian embryos. While glucose and lactate are consumed throughout the initial cleavages and rise in morulae and blastocysts [69], pointing towards a more glycolytic metabolism, the majority of ATP is produced by OXPHOS [63]. During the initial cleavages, mitochondrial metabolism does not change significantly compared to one of the mature MII, although a transient peak of oxygen consumption is observed shortly after fertilization, suggesting a possible increase in mitochondrial activity [70]. A sufficient level of mitochondrial activity is likely required for successful fertilization, as oocytes that fail to fertilize have significantly lower ATP levels and a lower mtDNA content [71,72]. Structurally, mitochondria in human embryos undergo changes in their shape during the transition between zygote and two-cell stages, and between the 4-cell to morula, and become progressively more elongated and the cristae assume the transversal distribution typical of condensed mitochondria [24].

When blastomeres start polarization and the blastocyst start forming, OXPHOS significantly increases, especially in trophectodermal cells, which have higher energy requirements as they are proliferating and must “prepare” for implantation. The inner cell mass (ICM) increases its OXPHOS levels later on, when cells start differentiating and proceed towards gastrulation [73].

Redox regulation in embryos have been investigated from several perspectives, and shown to be tightly related to the regulation of the cell cycle, acting as cycle regulators like MPF and other CDK-cyclin complexes [74]. In animal models, it has been shown how an increase in ROS levels right after fertilization, as observed for oxygen consumption, is associated with better embryo development, suggesting a possible interplay between calcium oscillation, activation of mitochondrial metabolism and redox regulation [70]. Furthermore, a rapid exposure of bovine embryos to H_2_O_2_ at the time of embryonic genome activation has been shown to promote embryo development, pointing towards a crucial role of redox balance in fertilization and the subsequent divisions [75]. Despite that, exact regulation of ROS during the physiological development of human embryos is unknown. Oxidative stress, on the contrary, impairs embryonic development in mice models where for example, high levels of ROS can cause embryonic arrest at the two-cell stage [74]. An important factor significantly influencing embryo development is oxygen tension. The level of oxygen in the mammalian female reproductive tract ranges from 1.5% to 8.5% O2, and in the endometrial environment it varies between 1.5% and 1.8% [76]. Considering this, another class of proteins that could play an important role in the redox balance of the embryo is the hypoxia inducible factors (HIFs). In normoxia (i.e., 20% O_2_), HIF are marked to degradation by prolyl-hydroxylases. In hypoxia, as in uterine cavity at the time of embryo implantation, active HIFs elicit a series of adaptive responses also involving mitochondria. These proteins are transcriptional regulators of hypoxic responses. They are heterodimeric proteins formed by two subunits: HIFβ, which is constitutively expressed, and HIFα, which presents three oxygen-dependent isoforms. Their activity is regulated by the abundance of α-ketoglutarate, a tricarboxylic acid cycle (TCA) metabolite [77], and changes in mitochondrial complexes II and III have been shown to stabilize HIFs. Hypoxia impacts morphology and protein aggregate formation in mitochondria, also causing changes in the electron transport chain (ETC) composition and activity. The adaptation of some of the complex of the ETC to hypoxia has been described to be mediated by HIFs. Additionally, modifications regulated by HIF in complex IV, such as changes in the enzymatic activity and inhibition by NO of the cytochrome c synthase (COX) [78], maintain ATP production under low oxygen levels together with a decreased formation of ROS, de facto preserving the integrity of the complex [79]. This highlights the strict relationship between oxygen levels, mitochondria and ROS, and their involvement in embryonic development.

The relatively low mitochondrial metabolism in early embryos has given rise to different hypothesis. In particular, one that caught the attention in recent years connects the very low metabolic rates to the protection from OS, and is named the “quiet embryo hypothesis”. This is based on different observations in humans and animal models, mainly regarding the depletion or supplementation of nutrients and their effects on embryo viability, which indicate how a reduced metabolism could protect from ROS induced DNA damage and apoptosis [80]. This was backed up also by studies in humans finding that excessive amounts of mtDNA in the cleavage-stage embryos and blastocyst never resulted in a successful pregnancy. However, the studies were subjected to controversies [81] and a direct link between a lower metabolism and an increased developmental competence in humans is lacking.

## 4. Mitochondrial DNA and OS

A unique feature of mitochondria is that they possess their own genetic material, the mitochondrial genome or mtDNA. Its origin is traced back to the prokaryotic nature of the organelle, which was incorporated in the eukaryotic cells during evolution, and therefore still retains its own genome. The human mtDNA, like bacterial genomes, is circular, relatively small (16.5 Kb) and with almost no introns. The small genome encodes for 13 proteins involved in OXPHOS, but most of the genes encoding for proteins involved in cellular respiration are encoded in the nucleus. This genetic transfer during evolution is speculated to be a mechanism of protection against mtDNA damage by the toxic byproducts of OXPHOS, as the species with a lower metabolic activity and lower level of ROS also possess the largest mitochondrial genomes, with a lower mutation rate than smaller ones, like the human mtDNA [82,83].

The problematic nature of the interaction between ROS and the mtDNA is reinforced by the fact that mitochondrial mutations can cause several detrimental effects in the respiratory chain, with a consequent increase in ROS presence. Different mutations in the mtDNA have been associated to increased ROS production, especially in complex I, as the mtDNA encodes for 7 subunits (NADH-ubiquinone oxidoreductase genes, ND1-ND6) of the 45 that form this complex. The insertion of an adenine in the ND5 gene (m.12417insA) has been shown to significantly increase the presence of superoxide, which generates H_2_O_2_ as an intermediate but it is not able to finalize its conversion to H_2_O and O_2_ [84]. Other mutations in ND5 (m.C12081A) increase the sensitivity of the cells to a mimetic of H_2_O_2_, 5-tert-butyl-hydrogen peroxide, which normally does not harm the cells, suggesting a higher basal activity of the scavenging machinery and a higher level of baseline OS. Similar findings have been described in cancer biology also for mutations affecting ND6 (m.G13997A and m.13885insC) and ND2 (m.G4776A) [85]. Analogous findings have been observed for mtDNA mutations in complex III [86] and IV [87] in other human diseases. Aside from pathological variants, also common single nucleotide polymorphisms (SNPs) found in some haplogroups (i.e., the set of homoplasmic variants that define individuals with a common geographical origin) have been associated to an increased production of ROS, as shown for variants of haplogroups N and M [88].

It is therefore clear that mtDNA mutations and OS are directly linked, and they likely act in a positive feedback loop, where the presence of one increases the other, and vice-versa.

## 5. Mitochondria, Oxidative Stress and Aging in Reproduction

The classic theory of biological aging was postulated decades ago, and it involves the progressive damage to tissues by the increasing presence of ROS generated by mitochondrial OXPHOS. The OS generated by ROS will damage the cells, especially the mitochondria, which will in turn slow down the production of ATP and generate a higher amount of ROS, up to the point where their concentration becomes too high and triggers cell death. Accordingly, tissues from older individuals have a defective mitochondrial network, with a reduced number of mitochondria, less mtDNA copy number, a higher frequency of mtDNA mutations and a lower production of ATP [89]. In mice, the increased presence of pathogenic point mutations and deletions in the mtDNA generates a progeroid phenotype, with a reduced fertility, again showing how mitochondrial dysfunction strictly associates to aging [90]. Additionally, a defective mitochondrial metabolism is associated to a higher production of ROS [91].

In reproduction, aging has a slightly different meaning, or at least it acts on a different time window. Biological aging is a continuous process, and manifests towards the end of the life cycle, while reproductive aging behaves differently in men and women. Fertility in men starts slowly declining around 45 years old, while in women it is observed earlier in life. At 35 years of age, fertility significantly speeds up its decline, and for this reason women older than 35 are considered of advanced maternal age (AMA). The causes of the fertility decrease are mainly the depletion of the ovarian reserve and a sharp increase in oocyte aneuploidy after 37 years of age. AMA is characterized by a reduction in the mitochondrial metabolism in the ovary, in the follicular environment and in the mature oocytes [92]. For example, at the follicular level, aging granulosa cells presents an impaired mitochondrial function, a decreased amount of mtDNA copies and an impaired biogenesis [93]. Again, the mitochondrial dysfunction is accompanied by a higher production of ROS. From knockout studies in mice, it seems also that decreasing the expression of antioxidant enzymes might induce features of premature aging, suggesting an important role in ROS scavenging in reproductive aging.

In oocytes, similar defects have been observed. Mitochondria in oocytes of AMA women present several structural changes, such as altered matrix density, swelling, abnormal cristae and an increased presence of vacuoles [94]. The altered morphology is mirrored by a decrease in their functionality, as measured by membrane polarization [95] and ATP production [96]. In addition, evidence shows a decreased amount of mtDNA and a reduced presence of mitochondrial replication factors, another hallmark of compromised mitochondrial regulation. In parallel, in transcriptomic studies, it has been observed that aging lowers the presence of antioxidant genes and upregulates gene related to oxidative stress, indicating an increase of ROS levels [97]. In mouse oocytes, increasing the concentration of ROS induces significant disruption of Ca^2+^ signaling, decreasing the fertilization rates and possibly altering mitochondrial physiology.

Together, all these observations mirror the ones from biological aging. However, some connections between these two different types of aging are missing. The “oxidative cost of reproduction” theory, for example, states that in order to reproduce, an organism must “pay” a price in terms of oxidative stress, reducing its lifespan. This might not be entirely correct, as a longer fertility window seems to extend the lifespan of the individuals in certain instances, rather than accelerate its aging [98].

Additionally, the mechanism connecting all the different features of aging in reproduction need to be elucidated: it is not known in fact if mitochondria can alone cause chromosomal abnormalities by providing an insufficient amount of ATP, or if this is mediated by the action of ROS, or if these features are just consequences of different processes induced by aging.

## 6. Mitochondrial OS during ART Procedures

In ART, procedures to obtain gametes and culture embryos are considered non physiological, and their potential effect on reproduction have been thoroughly investigated in mammalian models. To obtain a sufficient number of oocytes for an IVF (in vitro fertilization) cycle, for example, women have to undergo ovarian stimulation. The subsequent waves of follicle stimulating hormone (FSH), the suppression of the hypophysis and the other physiological modifications induced by hormonal treatments might increase cellular stress, and therefore OS might also be triggered, worsening the outcome of the cycle. Some studies in animal models already suggested that exposure to subsequent waves of hormones (FSH) and apoptosis during atresia in the ovary could lead to poor oocyte quality in females with advanced age [67,99]. Additionally, embryos derived from oocyte collected from ovaries that were repeatedly stimulated showed more mitochondrial aggregates. In the same line, Miyamoto and colleagues [100] reported that repeated ovarian stimulations in mice increase oxidative levels within the ovary and promote the accumulation of functionally impaired mitochondria, and also saw an abnormal distribution of the mitochondria in the oocytes. They also reported a reduction in the levels of mitochondrial Mn-SOD in the ovary. However, direct evidence of severe detrimental effects of ovarian stimulation in humans is lacking.

After fertilization, embryos are cultivated in vitro for three to five days. The time spent in the incubator is one of the most delicate steps in IVF, as there are several conditions that might negatively affect the quality of the embryo, like culture media composition, temperature, pH and oxygen tension.

Ma and colleagues [101] examined the levels of apoptosis and mitochondrial activity in a mouse embryo cultured under hypoxia or normal conditions, and found a higher mitochondrial membrane potential and lower levels of apoptosis in embryos cultured under hypoxic conditions [76]. This suggests, as previously mentioned, that HIFs are involved in the adaptation of the mitochondrial function and activity to hypoxia and play a physiological role in embryo development. During in vitro culture, in fact, human embryos are exposed to atmospheric oxygen concentration (20%). This exposure might impact HIFs function, therefore altering mitochondrial activity during early development. In mice, it was shown that a high oxygen concentration alters the mitochondrial structure and function in in vitro fertilized preimplantation embryos. Belli and colleagues [102] compared mitochondrial activity and morphology in mouse embryos recovered after spontaneous mating with in vitro fertilized embryos, cultured under 20% or 5% oxygen concentration. As expected, embryos from the uterus presented a better mitochondria number and function than embryos cultured in vitro (IVF-embryos) in all stages. They also found a decrease in the total number of mitochondria and an increase in abnormal shaped mitochondrial in the IVF-embryos compared with flushed embryos. Along the same lines, a blastocyst from the IVF-embryo groups presented higher levels of ROS, a decrease in the mitochondrial membrane potential and alteration in ATP levels, pointing out mitochondrial alterations. Importantly, these effects were more evident in the IVF-embryo group cultured under 20% oxygen than in the 5% group. In addition, significant differences in the mitochondrial membrane potential were found between the 20% and 5% IVF-group.

In summary, oxygen culture conditions impact mitochondria activity, morphology and function, affecting also ROS production, and this might participate in reducing the number of suitable embryos for use in ART.

Finally, another critical point is the effect that the culture media could have on the mitochondria and ROS production. Recently, the composition and proportions of each component were analyzed from 15 different culture media. The main energy source in the cleavage stage media were lactate and pyruvate, while glucose higher concentrations were found in the blastocyst stage medium. This correlates with the energetic requirements of the embryo at those different developmental stages [103].

Interestingly, the lactate to pyruvate ratio (L/P), often used to describe the balance between these two energy sources, greatly varied between the different media [104]. These significant differences might be affecting the activity of mitochondria, and consequently, ROS levels. However, further studies are needed to elucidate the contribution of culture media to OS levels, and their effects on embryo development.

During an in vitro culture, embryos secrete different metabolites to the culture media. Many studies have tried to find a relation between the metabolites detected in the spent media and the oxidative status of the embryo, and link it to a reproductive outcome. The study performed by Lee and colleagues [105] showed that the ROS levels found in follicular fluid and spent media is related to the embryo developmental capacity in different ways. Elevated ROS levels in the follicular fluid were related with poor quality embryos in day 3 after intracytoplasmic sperm injection (ICSI), but not after IVF, indicating that the ROS level in follicular fluid could be a good marker for oocyte competence in ICSI cycles. Higher levels of ROS in spent media on day 3 were correlated with embryo fragmentation, and a reduced implantation potential. However, these associations are not sufficient to prove the predictive value of ROS testing in follicular fluid and spent culture media.

## 7. Mitochondrial OS Testing in ART Outcomes

As we have seen, oxidative stress is caused by an imbalance between ROS generation and the antioxidant capacity of a specific cell. This imbalance could appear naturally by different factors, such as aging and lifestyle. In addition, we need to take into account its participation in the evolutionary theory about the oxidative costs of reproduction mentioned before [98,106,107].

Starting with the hypothesis of oxidative costs of reproduction and focusing on human reproduction, it has shown a transient increase of levels of lipid peroxidation, protein carbonyls and oxidative damage to blood lipids in pregnant women, when compared with nonpregnant women [108,109]. These studies indicate that human reproduction could lead to certain levels of oxidative stress, however the question remains open to understand whether this reproduction-induced oxidative stress could affect longevity or reproductive aging.

Two hypotheses have been formulated to explain those findings, a direct implication of metabolic rates in ROS production by mitochondria [110] and, on the contrary, reproductive-ROS production could be independent of the mitochondrial ROS increase [98]. In that sense, we need to take into account that diverse endogenous sources of ROS exist in eukaryotic cells, including mitochondrial oxidative phosphorylation and extramitochondrial enzymes (NOX) [111], imposing different OS costs.

ROS plays important roles in reproductive biology, but how can we identify its effect (physiological vs. pathological) on human fertility? Few studies have tested for the relationship between the total antioxidant capacity, ROS levels and OS markers on mammalian and human reproduction tissues [112,113,114,115].

As indicated previously, ART procedures might induce OS. For this reason, several groups tried to develop testing strategies to improve ART outcomes. This was done either by selecting cells with the lowest oxidative damage, or finding markers for a more accurate selection (Table 1).

At the moment, none of the studies have provided solid evidences of efficacy, and, as a whole, they are not conclusive, and more studies taking into account the physiological role of OS in human reproduction are needed.

## 8. ART Outcome Improvement through OS Management

Another field of extensive research is the development of treatments to reduce OS. Antioxidant supplementation has gotten popular in the past years to counteract the effects of ROS, either by increasing the scavenging or by improving mitochondrial activity. Current treatments tackle the problem from two sides mainly, the administration of an antioxidant treatment to the patient undergoing ART, or the supplementation of the culture media of the gamete/embryo, to reduce the OS levels in vitro.

The intake of Coenzyme Q10 (CoQ10) is one dietary supplementation that has captured the attention of researchers in different fields, and recently also in reproduction. One of this molecule’s roles, related to bioenergy in mitochondria, is transporting protons and electrons in the inner mitochondrial membrane [126]. As part of the mitochondrial system, CoQ10 plays a role in both male and female gamete physiology. Regarding seminal plasma and sperm, several studies have proven the positive effect of CoQ10 supplementation in men with idiopathic asthenozoospermia, reducing oxidative levels and increasing semen kinetics, therefore improving mitochondrial bioenergetics [127,128]. In the same line, the improvement of the mitochondrial function in an oocyte has been described after CoQ10 supplementation in an animal model with deficient CoQ10 production [129].

Marei and colleagues [130] have recently reported how the addition of an antioxidant that specifically targets the mitochondria, the Mitoquinone (MitoQ) [131] to the culture media, reduces oxidative stress and mitochondria uncoupling in embryos derived from compromised oocytes in an animal model.

Other chemicals such as resveratrol have also been tested. The addition of this supplement to oocyte maturation media were shown to improve oocyte developmental competence, improving mitochondrial function and increasing the number of mitochondria [132].

Melatonin has been used as an antioxidant to decrease mitochondrial ROS production in sperm by adding it to IVF media. It has been proven to be a powerful antioxidant in human spermatozoa by the inhibition of caspase activity, specifically capase-3 and caspase-9 [133]. However, based on the bovine model, it might seem to work in a concentration dependent manner. Addition of melatonin at low concentrations to IVF media induce changes in sperm motility, while at higher concentrations it seems to decrease the number of sperm with an intact acrosome and increase DNA damage [134]. On the other hand, melatonin media supplementation for intrauterine insemination (IUI) or IVF helps to protect sperm from ROS derived from the technique in an animal model [135].

Antioxidant production can be stimulated in a natural way by the reduction of O_2_ levels in an embryo culture. Ma and col. [101] demonstrated that reducing the levels under 3% O_2_ during embryo culture improves the expression of antioxidant enzymes that maintain mitochondrial ROS homeostasis, such as PRDX5 and Mn-SOD, and protects embryos by reducing ROS levels and apoptosis index in mice blastocysts.

Most of these antioxidants have been related to improved reproductive outcomes mainly in aged women. Although data seems promising, randomized control trials are still needed to prove the relationship between the antioxidant administration, either on the diet or culture media, and an improvement of the reproductive outcomes.

## 9. Conclusions

Oxidative stress, mainly caused by unbalanced ROS production, is being increasingly associated to alterations in the biology of reproductive cells. However, ROS have a prominent role in cellular processes, and emerged as mediators of the mitochondrial function in cellular signaling, and not only as toxic byproducts of OXPHOS. For this reason, a distinction between physiological ROS concentrations and OS is fundamental to discriminate between pathological conditions and normal fluctuations of ROS due to the metabolic shifts during gamete and embryo growth. To improve reproductive outcomes, OS testing and its management represents a promising tool, but further research is needed to identify the “roadmap” of metabolic cues characterizing appropriate gametes development, to be able to identify and select the most competent ones for infertility treatment.

## Figures and Tables

**Table 1:** Recent studies analyzing the redox status of (in)fertile people during assisted reproductive technologies (ART) cycles.

Cell Type	ASSAY	Method	Results	Ref.
Sperm	ORP	MiOXSYS	Higher values in infertile men	[116]
	SDF	Halosperm		[117]
Sperm	ORP	MiOXSYS	Higher values in infertile men	[118]
Sperm	GSH/GSSG	enzymatic	Altered in varicocele and leukocytospermia	[119]
	Catalase			[120]
Sperm	GSH-Px	enzymatic	Correlate with sperm parameters	[121]
Follicular fluid	HPSC		Levels of estradiol and progesterone are related to the redox status	[122]
Embryo	General Redox species	luminol	No association presence of ROS in culture media with embryo quality	[123]
Serum samples endometriosis	GSH/GSSG MDA TAC 8OHdG		Oxidative stress markers were good predictors of clinical pregnancy and live births after ICSI in women with stage I or II endometriosis.	[124]
Blood and Follicular fluid	ROS TAC	Flow cytometry Fluorometric assay	Oxidative stress markers were good predictors of ART outcomes.	[125]

ORP: oxidation–reduction potential; SDF: sperm DNA fragmentation; MDA: malondialdehyde; GSH/GSSG: Glutathione-disulphide glutathione; TAC: total antioxidant capacity; 8OHdG: 8-hydroxy-2’-deoxyguanosine; HPSC: hydrogen peroxide scavenging capacity.
